# Fréquence des anémies sévères chez les enfants âgés de 2 mois à 15 ans au Centre Mère et Enfant de la Fondation Chantal Biya, Yaoundé, Cameroun

**Published:** 2012-06-23

**Authors:** Félicitée Nguefack, David Chelo, Mathurin Cyrille Tejiokem, Angèle Pondy, Mina Julie Njiki kinkela, Roger Dongmo, Hubert Désiré Mbassi Awa, Jean Taguebue, Georgette Guemkam, Clémence Vougmo Meguejio Njua, Paul Olivier Koki Ndombo

**Affiliations:** 1 Centre Mère et Enfant (CME) de la Fondation Chantal Biya de Yaoundé, Cameroun; 2 Faculté de Médecine et des Sciences Biomédicales de l’Université de Yaoundé 1, Cameroun; 3 Centre Pasteur du Cameroun, Membre du Réseau International des Instituts Pasteur, Yaoundé, Cameroun; 4 Hôpital de district d’Efoulan, Cameroun

**Keywords:** Anémie sévère, fréquence, mortalité, enfant, mère, enfant, Cameroun

## Abstract

**Introduction:**

Les anémies sévères constituent une cause importante de décès d’enfants. Une analyse épidémiologique et clinique permettrait d’estimer la morbidité et mortalité y relatives afin lutter efficacement contre les causes.

**Méthodes:**

Notre étude rétrospective et descriptive porte sur les anémies sévères chez les enfants de 2 mois à 15 ans de juillet 2005 à juillet 2011. Les drépanocytaires et les enfants souffrant de néoplasie étaient exclus. Toutes les admissions de janvier 2008 à juillet 2011 et les décès totaux, qui répondaient aux critères ci-dessus ont été également répertoriés.

**Résultats:**

Ont été analysés 4735 cas d’anémie sévère dont 215 décès (4,5%). Entre janvier 2008 et juillet 2011, sur 12879 enfants hospitalisés 2456 souffraient d’anémie sévère dont 96 sont décédés, soit une mortalité spécifique de 0,7% et une létalité de 4,0%. Au total, 22,4% d’anémies sévères survenaient dans la tranche d’âge de moins de 12 mois. Celles de 12 à 59 mois et de plus de 5 ans représentaient respectivement 64,4% et 13,2% des cas. Le paludisme était l’étiologie évoquée chez 89,0% des cas, suivi du sepsis (9,4%). Les décès concernaient les enfants sévèrement anémiés âgés de 12 à 59 mois dans 67,2% de cas. La plupart de patients (84,8%) résidaient à Yaoundé (P = 0,004).

**Conclusion:**

Les anémies sévères restent fréquentes à Yaoundé. La mise en œuvre de da politique de gratuité des antipaludiques et l’utilisation des moustiquaires doivent être effectives. Le renforcement de ces mesures dès le début des saisons pluvieuses préviendrait les flambées d’anémies.

## Introduction

Dans les pays à ressources limitées, l’anémie sévère représente un risque important de mortalité [1]. Elle est associée à un taux d’occupation important des lits d’hôpitaux. Les anémies sévères sont une urgence médicale qui impose dans la majorité des cas des sanctions transfusionnelles sanguines dont les taux les plus élevés se retrouvent en Afrique subsaharienne. Le taux de transfusion était de 85,7% en 1999 dans un hôpital de Yaoundé et de 70% au Kenya [2, 3]. La charge de l’anémie palustre estimée en terme de DALYs (disability-adjusted life year) est très importante en Afrique où la mortalité est très élevée chez les enfants [4]. Si les problèmes d’anémie ne sont pas suffisamment pris en compte dans le programme de survie des enfants, il sera difficile que l’objectif du Millénaire pour le Développement (OMD) 4 soit atteint. Les causes sont multiples allant des infections aux déficits enzymatiques (G6PD) et en micronutriments, en passant par les parasitoses intestinales [5–7]. La situation est aggravée par le contexte d’infection au VIH [8, 9]. En effet le VIH contribue pour 45% de décès d’enfants ayant une anémie sévère [10]. D’après plusieurs études, le paludisme figure parmi les principales causes dans les zones d’hyper endémicité [7, 11]. L’anémie sévère fait partie des 15 critères de gravité du paludisme formulés par l’OMS [12]. Avec ou sans fièvre, les enfants ayant l’anémie modérée à sévère se recrutent surtout parmi ceux chez qui la parasitémie est importante, par rapport aux enfants exempts du plasmodium [13]. En Tanzanie, la décroissance du nombre de cas de paludisme était parallèle au déclin du taux d’anémie sévère [14]. Elle représentait 17% des hospitalisations chez les patients traités pour paludisme [15]. Les anémies sévères sont la conséquence d’un défaut de prise en charge correcte des cas simples de paludisme. Le risque de développer les signes de gravité telles que définis par l’OMS pour un paludisme non traité varient de 30 à 80% [16]. Le mécanisme physiopathologique est la destruction des érythrocytes ou la dysérythropoièse associée à certains facteurs étiologiques [17, 18].Une politique en faveur de la supplémentation en fer, le contrôle du paludisme permettrait de réduire l’incidence des anémies sévères et probablement les décès [19]. A ceci il faut ajouter l’amélioration de l’accès aux soins de qualité en général [20].

Nous rapportons dans cet article la fréquence et le profil évolutif des anémies sévères enregistrées au Centre Mère et Enfant de la Fondation Chantal BIYA (CME-FCB) durant les six dernières années.

## Méthodes

Une étude rétrospective descriptive a porté sur les registres des services des urgences et d’hospitalisations couvrant la période de juillet 2005 à juillet 2011 au CME-FCB. Cette formation sanitaire de référence est située dans la capitale politique du Cameroun (Yaoundé). Elle reçoit en majorité les populations de la ville de Yaoundé mais aussi des patients référés des structures sanitaires de petites agglomérations environnantes et d’autres régions du pays. Nous avons inclus dans cette étude tous les dossiers d’enfants reçus au cours de la période sus-indiquée âgés de 2 mois à 15 ans et chez lesquels un diagnostic d’anémie sévère avait été posé. Au sein du CME-FCB, on parle d’anémie sévère chez un enfant appartenant à la tranche d’âge retenue lorsqu’il présente soit un taux d’hémoglobine < 15%. Il peut également s’agir d’un cas dont le taux d’hémoglobine est compris entre 5 et 6 g/dl mais, chez qui on observe les signes cliniques d’intolérance notamment, la détresse respiratoire et/ou la tachycardie. Le paludisme grave est évoqué devant tout cas d’anémie sévère en contexte de fièvre survenue dans les 48 à 72 heures en l’absence de foyer infectieux bactérien évident. Dans la présente étude, les drépanocytaires et les patients souffrant des affections néoplasiques n’ont pas été inclus en raison de la conjonction de plusieurs facteurs à l’origine de l’anémie chez cette catégorie de patients. Les données collectées à partir des registres concernaient la date de consultation ou d’hospitalisation, l’âge, le sexe, le diagnostic, la date et le statut vital de l’enfant à la sortie. Les données manquantes étaient extraites des dossiers médicaux. Nous nous sommes également intéressés au nombre total de patients admis et de décès totaux enregistrés de janvier 2008 à juillet 2011, qui répondaient aux critères ci-dessus. Les paramètres analysés portaient sur les données sociodémographiques, la fréquence mensuelle des cas, le diagnostic étiologique probable, la durée d’hospitalisation et l’issue des malades.

Les données ont été analysées à l’aide du logiciel Epi info 2002, Version 2, January 30, 2003. L’étude a été approuvée sur les aspects scientifiques et éthiques par le comité de recherche biomédical institutionnel présidé par la direction du Centre Mère et Enfant de la Fondation Chantal Biya.

## Résultats

### Fréquences des anémies sévères parmi les hospitalisations au CME-FCB de 2008 à 2011

**Figure 1:**
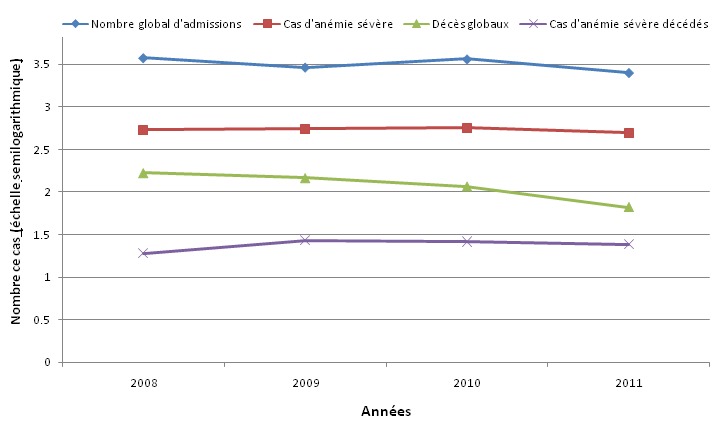
Evolution annuelle des hospitalisations, des anémies sévères, déces totaux et décès liés aux anémies sévères, Centre Mère et Enfant de la Fondation Chantal Biya, de Yaoundé, janvier 2008-juillet 2011

Entre janvier 2008 et juillet 2011, il y a eu 2456 cas d’anémies sévères et 96 décès sur un total de 12879 enfants hospitalisés, soit une mortalité spécifique de 0,7%, et une létalité de 4,0%. Les décès survenus chez les enfants anémiés représentaient 19% des décès totaux enregistrés durant cette période. La Figure 1 présente l’évolution annuelle de l’ensemble des admissions, décès totaux, des cas d’anémie sévère et décès y relatifs sur une période de trois ans six mois. L’évolution de la proportion d’enfants hospitalisés annuellement était croissante, alors que celle des anémies sévères était restée constante, soit 25-26% en 2008-2010 (X^2^, de tendance = 483,4 P < 10^-4^). Les décès chez les patients anémiés augmentaient au contraire; de 20% de décès enregistré en 2008, on est passé à 28% puis 27% en 2009 et 2010 respectivement. Cette mortalité était plus marquée durant les six premiers mois de 2011 (25% de décès). A l’opposé, la tendance des décès parmi l’ensemble des enfants hospitalisés étaient en baisse régulière (X^2^, de tendance = 17,1 P < 10^-4^).

### Analyse des anémies sévères enregistrées au CME de juillet 2005 à Juillet 2011

Au cours de cette période, 4735 cas d’anémie sévère et 215 décès ont été enregistrés, soit une létalité de 4,5% proche du chiffre ci dessus.

### Caractéristiques des enfants anémiés inclus dans notre étude

L’âge médian des enfants était de 21 mois avec un écart interquartile de 24 mois. Les enfants de sexe masculin représentaient 55,9% (Tableau 1). Au total, 22,4% d’anémies survenaient dans la tranche d’âge de moins de 12 mois. Les enfants âgés de 12 à 23 mois, ceux de 24 à 59 mois ainsi que la tranche de plus de 5 ans représentaient respectivement 30,0%, 34,4% et 13,2% des cas. Chez 89% de patients anémiés, le diagnostic étiologique évoqué était le paludisme, puis suivait le sepsis (9,4%). La plupart de décès (71,3%) sont survenus en début d’hospitalisation avec un délai médian entre l’hospitalisation et le décès d’un jour (Tableau 2). Il a affecté les filles et les garçons dans les mêmes proportions (respectivement 4,9% et 4,1% de garçons et de filles anémiés). Le paludisme était également le plus retrouvé chez les décédés (81,9%), tandis que le sepsis et le SIDA y contribuaient respectivement pour 12,1% et 1,9% (p= 10^-4^). Le décès a concerné majoritairement les plus jeunes enfants sévèrement anémiés (p = 0,005). Il est survenu chez 10,1%, 3,7% et 5% des cas d’anémies sévères âgés respectivement de 2 à 23 mois, 24 à 59 mois et de plus de 60 mois. Ces tranches d’âge représentaient respectivement 57,7%, 27,9% et 14,3% des décès chez les anémiés (Tableau 2). En ce qui concerne la provenance des patients, selon qu’ils résidaient hors de Yaoundé ou dans cette ville, le décès survenait chez 4,6% et 4,5% des cas respectivement. La plupart de patients (83,5%) résidaient à Yaoundé (P = 0,007), seul 16,5% de cas provenaient d’ailleurs et les décès survenus dans ce dernier groupe représentait la même proportion (soit 16,7% des décès totaux)

**Tableau 1:** Répartition des anémies sévères selon le sexe et la tranche d’âge

	Sexe		
Groupes d’âge (ans)	Féminin (N = 2090) n (%)	Masculin (N= 2645) n (%)	Total (N= 4735) n (%)
0-1	480 (23,0)	581 (22,0)	1061 (22,4)
1-2	610 (29,2)	812 (30,7)	1422 (30,0)
2-5	737(35,3)	892 (33,7)	1629 (34,4)
>5	263 (12,6)	360 (13,6)	623 (13,2)

**Tableau 2:** Caractéristiques des patients hospitalisés pour anémie sévère (juillet 2005-juillet 2011)

Variables n (%)	Favorable n (%)	DCD n (%)	Sortie exigée n (%)	Transféré n (%)	Total	p
**Sexe**						
Masculin	2339 (88,4)	130 (4,9)	147 (5,9)	29 (1,1)	2645 (100)	0,57
Féminin	1864 (89,2)	85 (4,1)	119 (5,7)	22 (1,1)	2090 (100)	
**Résidence**						
Yaoundé	3533 (89,3)	179 (4,5)	207 (5,2)	36 (0,9)	3955 (84,8)	0,007
Hors de Yaoundé	670 (86,0)	36 (4,6)	59 (7,6)	14 (1,8)	779 (15,2)	
**Age**						
Médiane (EIQ)	21 (24)	19 (25)	18 (19)	24 (61)	21 (24)	
**Age en classes**						
0-1	917 (86,4)	61 (5,7)	69 (6,5)	14 (1,3)	1061 (100)	0,005
1-2	1262 (88,5)	63 (4,4)	87 (6,1)	10 (0,7)	1422 (100)	
2-5	1474 (90,5)	60 (3,7)	82 (5,0)	13 (0,8)	1629 (100)	
>5	550 (88,3)	31 (5,0)	28 (4,5)	14 (2,2)	623 (100)	
Durée séjour Médiane	5 (3)	1 (1)	2 (4)	2,5 (4)	5 (3)	
**(EIQ) en classes (jours)**						
1	41 (1,0)	154 (71,3)	98 (37,0)	18 (36,0)	310 (6,6)	<10-4
2-5	2173 (51,8)	51 (23,7)	114 (43,0)	21 (42,0)	2359 (49,9)	
>5	1980 (47,2)	11 (5,1)	53 (20,0)	11 (22,0)	2055 (43,5)	
**Etiologies**						
Paludisme grave	3760 (89,3)	176 (4,2)	234 (5,6)	41 (1,0)	4211 (100)	<10-4
Sepsis	390(87,6)	27 (5,8)	21(4,7)	8 (1,8)	445 (100)	
Malnutrition sévère	34(63,0)	9(16,7)	9(16,7)	2 (3,7)	54 (100)	
SIDA	15(71,4)	4(19,0)	2(0,7)	0 (0,0)	21 (100)	
Autres	2(0,0)	0(0,0)	0(0,0)	0 (0,0)	2 (100)	

EIQ : écart interquartile (25-75%)

### Evolution annuelle des cas d’anémie sévère et des décès de juillet 2005 à juillet 2011

**Figure 2:**
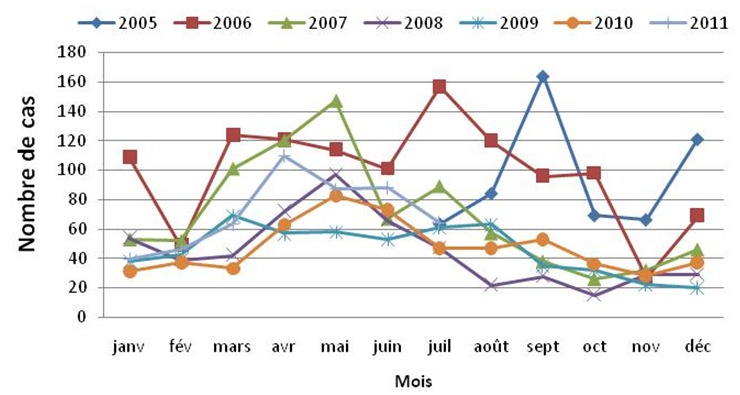
Fréquence mensuelle et annuelle des cas d’anémies sévères, Centre Mère et Enfant de la Fondation Chantal Biya, de Yaoundé, juillet 2005 à Juillet 2011

**Figure 3:**
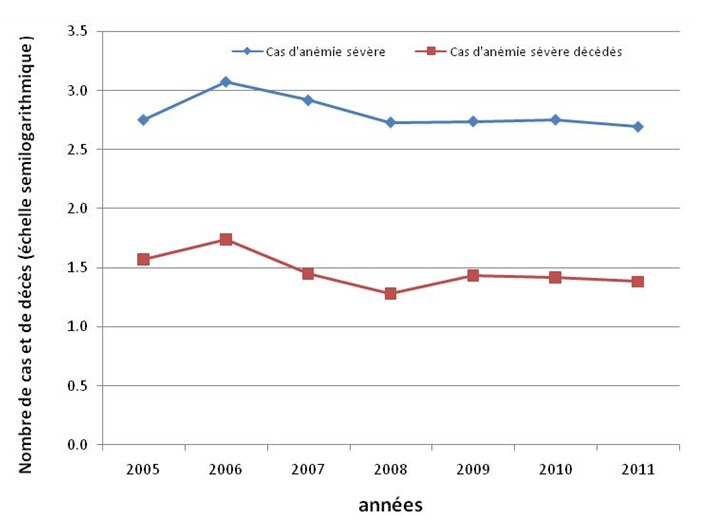
Evolution annuelle des cas d’anémies sévères et de déces y afférents, Centre Mère et Enfant de la Fondation Chantal Biya, de Yaoundé, juillet 2005 à Juillet 2011

La fréquence des anémies était très élevée en 2005 et en 2006. C’est ainsi que 567 et 1185 cas étaient enregistrés en six mois (juillet-décembre 2005) et en 2006 respectivement, représentant 12% et 25% de l’ensemble des 4735 cas (Figure 2). A partir d’avril 2007, les anémies sévères avaient diminué sensiblement, passant de 17% à 12%. Ces cas d’anémies évoluaient selon une saisonnalité, les pics de haute fréquence s’observant entre mars et juin, suivis d’une décroissance pour atteindre un bas niveau vers la fin de l’année. La courbe constamment basse s’est observée en 2009. Par la suite, la tendance était en faveur d’une augmentation du nombre de cas car, en 2011 on a noté en six mois seulement, une fréquence de 10%, proche des chiffres obtenus annuellement au cours des trois années précédentes (X^2^ de tendance = 48,7; P = 10^-4^). Pour ce qui est des décès, l’évolution annuelle suivait la courbe des cas (Figure 3). Les garçons représentaient 55,9% des cas.

## Discussion

L’anémie sévère était la quatrième cause de consultation au service d’urgence du CME-FCB (7,7%), et contribuait pour 56,34% des décès en l’an 2000 [21]. Nos résultats montrent que malgré la diminution des cas, par rapport à ce qui s’était produit au début de l’étude, les anémies demeurent un problème de santé majeur. La tendance des décès en général était en baisse alors que celle des anémies sévères était constante et les décès y relatifs en hausse. Cette situation s’observe en dépit des grands programmes de santé susceptibles de couvrir l’essentiel des problèmes de santé des enfants. L’analyse des différentes courbes permet de conclure en référence à celle de 2009, qu’il y aurait un relâchement dans la mise en œuvre des interventions en matière de lutte contre les principales causes de l’anémie et les décès y relatifs. Cette diminution des cas en 2009 reflèterait la baisse générale du nombre total d’enfants admis durant cette période. C’est ainsi que l’augmentation de la fréquentation du CME-FCB qui s’en est suivie s’est faite avec les cas d’anémie sévère. Etant donné son statut humanitaire avec la pratique des coûts de soins bas et sa qualité d’hôpital de référence, les anémies sévères y seraient référées préférentiellement par d’autres formations sanitaires. En outre, d’autres facteurs tels que l’accessibilité géographique, financière et médicamenteuse ainsi que la préférence des parents auraient influencé l’utilisation de ce Centre Pour la prise en charge d’autres affections. Ceci n’explique pas cependant le nombre élevé de décès en rapport avec les anémies, et qui nécessite que d’autres interventions soient mises en place pour prendre en charge les anémies sévères en situation critique dont la plupart décèdent à l’admission.

Les causes des anémies sévères sont multifactorielles dans les pays en voie de développement [5, 6, 7]. Sa fréquence était élevée entre avril-mai, coïncidant ainsi avec la saison de forte pluviosité observée dans la région du centre-Cameroun où se trouve le Centre d’étude. Comme dans la présente étude, plusieurs autres ont incriminé le paludisme comme étant la principale étiologie [15, 22–24]. Au Ghana, le taux d’anémie était élevé (22,1%) pendant la saison pluvieuse et correspondait à la haute transmission du paludisme [25]. Yaoundé est située dans la région du centre, une zone forestière dite de transmission continue où le paludisme sévit selon un mode endémique et saisonnier. Une étude réalisée dans cette ville en 2008, chez les enfants âgés de 5 à 10 ans a permis de conclure que la drépanocytose et le paludisme jouaient un rôle primordial dans la survenue des anémies [26]. Le paludisme représentait 71,58% des étiologies en 2003 au CME-FCB [21]. Le taux d’anémie sévère serait plus élevé dans notre étude, si nous avions inclus les drépanocytaires. Il a été démontré qu’une forte parasitémie était associée à une anémie et un taux de mortalité élevé chez ce groupe de malades [27]. D’autres auteurs par contre, pensent qu’il y a surestimation du paludisme chez les patients fébriles résidant dans des zones de faible transmission du paludisme [28]. En effet, ils n’ont pas trouvé de différence significative entre les anémies sévères chez les patients avec ou sans plasmodium dans le sang [28].

Chez les enfants gabonais, la tranche d’âge de un à 11 mois avait un risque élevé de présenter une anémie sévère d’origine palustre. Par contre ceux de plus de six ans étaient susceptibles de faire une anémie sévère d’étiologie autre que le paludisme [29]. Le risque d’anémie sévère d’origine palustre était élevé chez les nourrissons d’un an en période de haute intensité de transmission et chez ceux de 2 ans, en période de basse intensité [30]. L’anémie est ainsi considérée comme un indicateur utile pour estimer la charge morbide du paludisme dans les zones de forte endémicité [31, 32].

Certains enfants seraient porteurs d’anémie asymptomatique. Une étude réalisée chez les enfants Tanzaniens a permis de retrouver dans la communauté, des enfants qui avaient un taux d’hémoglobine bas, c′est-à-dire < 8 g/dl et < 5 g/dl respectivement chez 87%, 39% et 3% d’enfants [33]. Les auteurs l’ont qualifiée « d’anémie silencieuse ». Par ailleurs, même quand les symptômes apparaissaient, ils n’orientaient pas toujours vers une gravité de la pathologie [33]. Au sud Cameroun, globalement la prévalence des anémies était élevée (47%) chez les nourrissons de moins de six mois [34]. Au Nigéria, elle a affecté 4,16% d’enfants âgés de 7 mois à 12 mois [35]. Dans ces conditions, la survenue d’événements aigus tels que le paludisme et les infections bactériennes précipiterait le statut hématologique déjà fragilisé chez ces enfants.

La quasi-totalité de ces cas d’anémie sévère ont été transfusés, le taux de transfusion sanguine était de 87% dans une autre étude réalisée à Yaoundé [2]. La transfusion sanguine représente une protection contre certains décès (1). Cette mesure salvatrice n’est pas toujours facile à mettre en œuvre en pratique courante, elle n’est pas non plus dénuée de risques réactionnels et de transmission de maladies [36]. En effet des difficultés se posent par rapport à la disponibilité du sang en qualité et en quantité; la recherche de certains agents infectieux n’étant pas systématique [11]. En Tanzanie les anémies sévères auraient exposé au VIH environ 19 000 enfants ayant survécus du fait de la transfusion sanguine [15]. Certains états septiques post transfusionnels seraient la conséquence de la contamination bactérienne du sang transfusé. En effet les bactéries ont été isolées dans 8,8% des poches de sang total destines à la transfusion au Kenya [37]. Sa vulgarisation est en général proscrite même dans des situations critiques par certains auteurs, son bénéfice étant très limité [38]. La transfusion sanguine doit être réservée aux cas où l’anémie sévère s’accompagne des signes de d’intolérance à l’exemple de la détresse respiratoire et surtout en début d’hospitalisation [39]. Considérant son risque potentiel, il est important de lutter contre tous les facteurs qui prédisposent à l’anémie.. L’accent est mis sur l’effet bénéfique du traitement du paludisme simple pour prévenir les formes sévères et l’anémie d’origine palustre [40]. Certaines études ont démontré les effets protecteurs du traitement préventif intermittent utilisant les ACT sur le paludisme et l’anémie [41].

Au Cameroun, le plan stratégique national de lutte contre le paludisme [42] met l’accent sur la promotion de l’utilisation de la moustiquaire imprégnée d’insecticide (MII) dont la distribution suit son cours sur toute l’étendue du territoire. Depuis 2010, le gouvernement camerounais offre gratuitement les ACT pour le traitement du paludisme simple chez les enfants de moins de 5 ans. Ces mesures permettraient de lever certaines barrières (géographique, financière, médicamenteuse) à l’accès aux soins. Malheureusement, leur application serait ineffective du fait de l’accessibilité limitée aux MII et du mythe qui entoure leur utilisation. En effet une faible proportion d’enfants de moins de cinq ans dormait sous MII selon les résultats de l’EDSIII [43].

Quant aux décès survenus chez les patients anémiés, trois « retards » en cause seraient évités. Le premier concerne l’identification de la profondeur de la pâleur palmaire et certains signes de gravité par les parents ou l’agent de santé. Le deuxième est l’urgence de la prise de décision et la consultation ou la référence dans une formation sanitaire appropriée. Enfin, la décision de transfusion sanguine basée sur les évidences clinques dans de délais et des conditions appropriés. La stratégie de « Prise en Charge Intégrée des Maladies de l’Enfant» dans sa composante communautaire renforce les capacités des parents et des familles à pouvoir mettre en œuvre les deux premiers points [44]. Afin d’améliorer la sensibilité de cette stratégie, certains auteurs recommandent que la recherche de l’anémie prenne en compte aussi bien la pâleur conjonctivale, le geignement et la pâleur palmaire [45].

La malnutrition, est un également un facteur essentiel contribuant à la morbidité et à l’anémie palustre. Le programme de lutte contre le paludisme à lui seul ne pourrait avoir un impact considérable sans le programme nutritionnel [46]. Par ailleurs, une prévention primaire des anémies ferriprives et du paludisme, permettrait de réduire substantiellement les décès liés aux anémies chez les jeunes enfants vivant dans les zones impaludées [4]. La variation du taux d’hémoglobine avec l’âge dépendrait aussi des conditions qui ont précédé la naissance de l’enfant; c’est ainsi que le faible poids de naissance et l’anémie fœtale sont associés à un faible taux d’hémoglobine dans la période infantile [47].

Cette évaluation présente néanmoins quelques limites. L’étude était basée à l’hôpital dont la population étudiée ne serait pas représentative de la ville de Yaoundé en raison des paramètres importants pouvant contribuer au choix du site (accessibilité géographique, financière et préférence). La position de ce centre comme référence peut contribuer également à la concentration des cas sévères. L’aspect présomptif des diagnostics enregistrés dans le cadre de cette étude est également un autre élément majeur à prendre en compte, certains de nos patients pouvaient avoir des tares connus (Hb SS, maladie lymphoproliférative). Nous n’avons pas pris en compte les drépanocytaires dans nos analyses, du fait de la complexité de la physiopathologie de l’anémie sur ce terrain. Malgré tout, cette étude a l’avantage d’apporter des éléments de base pouvant permettre de mieux bâtir des travaux plus spécifiques sur cette thématique.

## Conclusion

Les anémies sévères représentent une charge morbide importante dans notre contexte. Les enfants âgés de moins de 12 mois et ceux de 12 à 59 mois représentaient respectivement 22,4% et 67,2% des cas). Le paludisme était le facteur prépondérant évoqué dans les étiologies de ces anémies. Le nombre de décès annuels chez les enfants anémiés sévèrement était élevé et en augmentation par rapport aux décès globaux. La plupart survenaient beaucoup plus dans les premières 24 heures d’admission et traduisent la gravité du tableau clinique et le retard de la prise en charge. La mise en œuvre de la « Prise en Charge Intégrée des Maladies de l’Enfant au niveau communautaire constitue une des solutions à ce problème. Elle permettrait non seulement de renforcer les connaissances des parents et des familles, mais aussi de mettre à leur disposition les outils permettant de prendre en charge de façon appropriée les enfants malades. Des actions de sensibilisation sur l’utilisation des services de santé dès les premiers signes de la maladie doivent être renforcées. Enfin, la mise en œuvre effective de da politique gouvernementale de la gratuité des ACT chez les enfants et l’utilisation des moustiquaires imprégnées d’insecticide contribuerait à réduire considérablement l’incidence des anémies palustres. Si ces actions sont renforcées entre février-mars, elles préviendraient des flambées en période pluvieuse.
